# Resident-, prescriber-, and facility-level factors associated with antibiotic use in long-term care facilities: a systematic review of quantitative studies

**DOI:** 10.1186/s13756-024-01385-6

**Published:** 2024-03-06

**Authors:** Aurélie Bocquier, Berkehan Erkilic, Martin Babinet, Céline Pulcini, Nelly Agrinier, on behalf of the ORANEAT Study Group

**Affiliations:** 1 Université de Lorraine, Inserm, INSPIIRE, Nancy, F-54000 France; 2 CHRU-Nancy, INSERM, Université de Lorraine, CIC, Epidémiologie clinique, Nancy, F-54000 France; 3 Centre régional en antibiothérapie du Grand Est AntibioEst, Université de Lorraine, CHRU-Nancy, Nancy, F-54000 France

**Keywords:** Anti-bacterial agents, Nursing homes, Long-term care, Epidemiologic factors, Systematic review

## Abstract

**Background:**

Antimicrobial stewardship programmes are needed in long-term care facilities (LTCFs) to tackle antimicrobial resistance. We aimed to identify factors associated with antibiotic use in LTCFs. Such information would be useful to guide antimicrobial stewardship programmes.

**Method:**

We conducted a systematic review of studies retrieved from PubMed, Cochrane Library, Embase, APA PsycArticles, APA PsycINFO, APA PsycTherapy, ScienceDirect and Web of Science. We included quantitative studies that investigated factors associated with antibiotic use (i.e., antibiotic prescribing by health professionals, administration by LTCF staff, or use by residents). Participants were LTCF residents, their family, and/or carers. We performed a qualitative narrative synthesis of the findings.

**Results:**

Of the 7,591 screened records, we included 57 articles. Most studies used a longitudinal design (*n* = 34/57), investigated resident-level (*n* = 29/57) and/or facility-level factors (*n* = 32/57), and fewer prescriber-level ones (*n* = 8/57). Studies included two types of outcome: overall volume of antibiotic prescriptions (*n* = 45/57), inappropriate antibiotic prescription (*n* = 10/57); two included both types. Resident-level factors associated with a higher volume of antibiotic prescriptions included comorbidities (5 out of 8 studies which investigated this factor found a statistically significant association), history of infection (*n* = 5/6), potential signs of infection (e.g., fever, *n* = 4/6), positive urine culture/dipstick results (*n* = 3/4), indwelling urinary catheter (*n* = 12/14), and resident/family request for antibiotics (*n* = 1/1). At the facility-level, the volume of antibiotic prescriptions was positively associated with staff turnover (*n* = 1/1) and prevalence of after-hours medical practitioner visits (*n* = 1/1), and negatively associated with LTCF hiring an on-site coordinating physician (*n* = 1/1). At the prescriber-level, higher antibiotic prescribing was associated with high prescription rate for antibiotics in the previous year (*n* = 1/1).

**Conclusions:**

Improving infection prevention and control, and diagnostic practices as part of antimicrobial stewardship programmes remain critical steps to reduce antibiotic prescribing in LTCFs. Once results confirmed by further studies, implementing institutional changes to limit staff turnover, ensure the presence of a professional accountable for the antimicrobial stewardship activities, and improve collaboration between LTCFs and external prescribers may contribute to reduce antibiotic prescribing.

**Supplementary Information:**

The online version contains supplementary material available at 10.1186/s13756-024-01385-6.

## Introduction

Antimicrobial resistance (AMR) is one of the top 10 global health threats according to the World Health Organisation (WHO) [1, 2]. AMR, notably bacterial resistance to antibiotics, is of particular concern in long-term care facilities (LTCFs) where antibiotic use is frequent and often unnecessary or inappropriate [3] and antibiotic-resistant bacteria more prevalent than in the community [4].

Antimicrobial stewardship (AMS) (i.e., a coherent set of actions which promote using antimicrobials responsibly [5]) has proven effective in reducing antimicrobial use (in particular broad-spectrum antibiotics), prevalence of multidrug resistant organisms (MDRO), and total costs of care in different healthcare settings [6–8]. Evidence suggests greater effectiveness in intensive care units [8] and in paediatric care settings [6] than in LTCFs for which evidence is scarce, inconsistent, and of low quality [9–11]. The AMS interventions in LTCFs mainly involved multiple components, most often educational strategies and promotion of clinical guidelines targeting both physicians and nurses; few interventions used tailored strategies adapted to the local LTCF context [9, 10]. Besides, studies that have conducted a process evaluation showed that adoption of interventions by facilities and staff was low [10].

LTCFs are indeed challenging settings for AMS implementation [12]. Due to less specific symptoms, comorbidities, and impaired ability to report symptoms, the diagnostic process is often complex in LCTF residents. Moreover, LCTFs often face staff turnover and absenteeism, and a lack of resources dedicated to quality monitoring and improvement [12]. All of these specificities combined hinder implementation of AMS programmes. In addition, specific determinants of antibiotic use in LCTFs might have been overlooked in the development of AMS programmes so far. This might explain part of the inconsistent evidence about AMS programme effectiveness in LCTFs [9–11].

One meta-synthesis of qualitative studies highlighted that specific contextual factors (e.g., restricted access to on-site resources) and social factors (e.g., nurses’ central role) critically impact antibiotic prescribing in LCTFs [13]. However, to date, to the best of our knowledge, no study has reviewed the empirical evidence derived from quantitative studies identifying factors significantly associated with antibiotic use in LCTFs.

Therefore, we conducted a systematic review of quantitative studies to identify and summarise the factors associated with antibiotic use among residents in LTCFs.

## Methods

### Search strategy

We followed the Preferred Reporting Items for Systematic reviews and Meta-Analyses (PRISMA) 2020 statement guidelines (see checklist in Table S1) [14], and registered the protocol with the International Prospective Register of Systematic Reviews (PROSPERO) database (registration number CRD42022345784).

We identified peer-reviewed journal articles published from inception until the 24th of May, 2022 on the following databases: MEDLINE (PubMed), Cochrane Library, Embase, APA PsycArticles, APA PsycINFO, APA PsycTherapy, ScienceDirect, and Web of Science. We also crosschecked reference lists of all included articles.

We used keywords and MeSH terms at the intersection of three topics: “antibiotic use”, “LTCF”, and “factors/determinants” (see search strategies in Table S2). For the purpose of this review, LTCFs were defined as structures accommodating dependent yet medically stable older adults and providing adapted medical and paramedical care in a permanent or temporary arrangement [15]. They typically provided 24-hour supervision and a high level of nursing care, but did not provide specialised medical care or invasive medical procedures.

### Eligibility criteria

Studies were eligible for inclusion if they met the following criteria. First, study population of eligible studies included either LTCF residents, their family (i.e., people who are related to the residents, such as their children, nephews/nieces, brothers/sisters), and/or carers, especially all health professionals who may play a role in antibiotic use among LTCF residents. Second, eligible studies focused on exposure to factors of any kind, as for example factors related to patients, health professionals, or LTCFs’ characteristics. Third, eligible studies included any outcome related to antibiotic use: e.g., antibiotic prescribing by health professionals (volume or inappropriateness, e.g. prolonged treatment duration), administration by LTCF staff, use (including self-medication) by residents. Fourth, eligible studies relied on the following design: ecological, cross-sectional, case-control, or longitudinal studies quantitatively assessing associations between exposure and outcome. We excluded literature reviews and studies that addressed effectiveness of a formal AMS programmes as their primary objective.

After the removal of duplicates, two reviewers (among B.E. M.B., and A.B.) independently screened articles based on titles and abstracts. Then two reviewers (among B.E., M.B., A.B., and N.A.) independently screened retrieved full texts against the eligibility criteria. The screening process used the free web application Rayyan [16]. Differences between authors were resolved through consensus and the opinion of a third reviewer when needed.

### Data extraction

Two reviewers (A.B. and B.E.) independently performed data extraction for ten articles, and solved discrepancies by consensus. The remaining articles were divided in two batches, and each reviewer extracted data for all articles in one of the two batches.

The following information was extracted: study characteristics (first author, year of publication, journal, study country, year/period of data collection, study objective, design and sample size), participants’ characteristics, type of statistical analysis, outcome measures (nature, definition, unit, data collection method), and the list of the factors investigated (nature, definition, and unit). For each factor that was statistically significantly (i.e., *p* ≤ 0.05) associated with at least one outcome, we extracted the main findings (direction and magnitude of the association, and p-value).

### Quality assessment

We assessed studies for quality of reporting using a purpose-built standardised tool adapted from the Strengthening the Reporting of Observational Studies in Epidemiology (STROBE) statement checklist [17]. We included 16 items assessing the quality of reporting of the study design, setting, eligibility criteria for participants, sample size, variables, data source, method of measurement, and statistical methods (Table S3). For each study, based on the extracted data, the reviewer coded each item 0 (poor quality of reporting) or 1 (good quality of reporting) and then calculated the study score by summing the 16 items’ code (theoretical range: 0 for minimal quality of reporting to 16 for maximal quality of reporting).

### Data synthesis

First, we conducted a descriptive synthesis of studies’ characteristics (e.g., design, type of outcomes, and factors investigated).

Second, due to heterogeneity pertaining to selected outcomes, metrics of the factors investigated, and the methods used across studies, we could not perform a meta-analysis, and thus provided a qualitative narrative synthesis of the findings. To make interpretation easier, relying on thematic content analysis, we grouped the factors investigated in the included studies into categories and sub-categories, defined by consensus between researchers involved in the review and derived from classifications of determinants of antibiotic use applied in other settings (e.g., in primary care settings) [18–20]. We identified five categories of factors: resident-level, prescriber-level, prescription-level, facility-level and other contextual factors (e.g., year, season).

For each category of outcome and factor identified, we relied on the number of studies that found a statistically significant positive association (i.e., *p* ≤ 0.05), a statistically significant negative association (or both types of association depending on the outcome for example), and the number of studies that found no statistically significant association. For each factor, if > 50% of the studies which investigated the factor showed a statistically significant association in the same direction, we used the term “trend toward” to summarise the results in the Results section; in other cases, we used the term “mixed results”. For each factor with a trend, we described five of the Hill’s criteria for causation [21]. We assessed temporality using the number of longitudinal studies over the number of studies that found a statistically significant association consistent with the trend. We also assessed strength (e.g., RR, OR), and dose-response relationship (coded as ‘yes’ if a dose–response relationship was observed in at least one study; ‘no’ if there was no dose–response relationship in any study or ‘not applicable’ if the factor metrics did not allow to measure a dose-response relationship). Then we assessed internal consistency (i.e., number of studies that used adjusted analyses over the number of studies that found a statistically significant association consistent with the trend) and reproducibility (i.e., the number of studies that found a statistically significant association consistent with the trend over the total number of studies that investigated the concerned factor).

## Results

### Study selection and characteristics

**Fig. 1:**
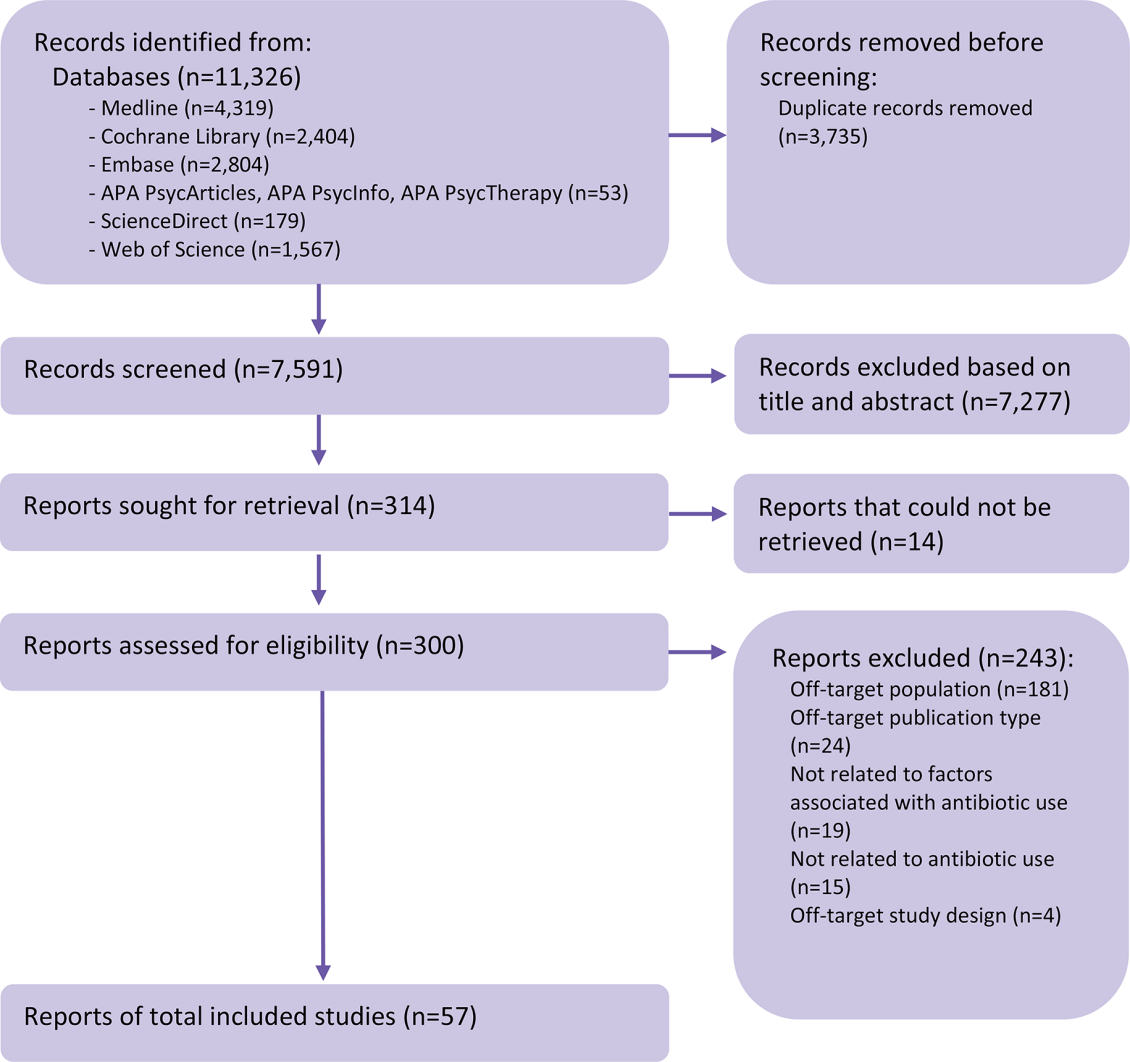
PRISMA 2020 flow diagram for the systematic review of quantitative studies on the factors associated with antibiotic use in LTCFs LTCF, long-term care falicity

We identified 11,326 records. After removing duplicates, we selected 300 articles based on title and abstract, of which 57 [19, 22–77] met the inclusion criteria after full-text reading (see the flow diagram in Fig. 1, the list of full-text reports that could not be retrieved or excluded in Table S4, and the list of the included articles in Table S5).

**Fig. 2:**
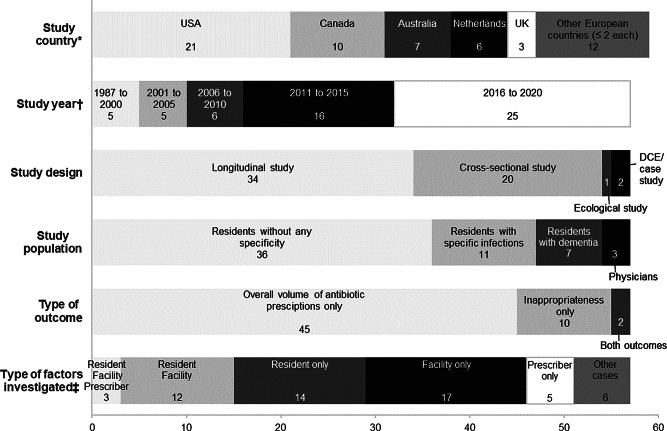
Main characteristics of selected studies about factors associated with antibiotic use (*n* = 57) DCE, Discrete Choice Experiment; UK, United Kingdom; USA, United States of America. * One study included data from the USA and from Canada and one study included data from the USA and the Netherlands. † Last year of data collection. ‡ All in all, 32 studies investigated resident-level factors, 32 facility-level factors, and 8 prescriber-level factors.

Characteristics of the included studies are described in Fig. 2 and Table S6. Most studies used data collected in North America (30/57), and a longitudinal (34/57) or cross-sectional (20/57) design. Adjusted statistical analyses were performed in 35/57 studies (Table S6). The quality of reporting score ranged from 7 to 15 (median = 12). Only 8/57 studies reported any efforts to address potential sources of bias and 7/57 how missing data were handled (Table S3).

Studies included two main types of outcome. First, overall volume of antibiotic prescriptions (*n* = 45/57), at the resident-level (e.g., being prescribed an antibiotic on the index assessment date, antibiotic days of therapy per 1,000 resident days) or aggregated at the facility-level (number of antibiotic courses started per 1,000 resident care days per month, total antibiotic prescriptions in defined daily doses per 100 bed-days). Second, inappropriate antibiotic prescription (*n* = 10/57) (e.g., in terms of antibiotic drug indication, choice, dose, and/or treatment duration). Two studies included both types of outcomes.

Factors investigated in the included studies related either to the resident, the prescriber, the LCTF’s characteristics, or to other contextual factors (e.g., year, season). Only three studies investigated simultaneously resident, prescriber, and facility-level factors (Fig. 2).

### Factors associated with overall volume of antibiotic prescriptions

**Fig. 3:**
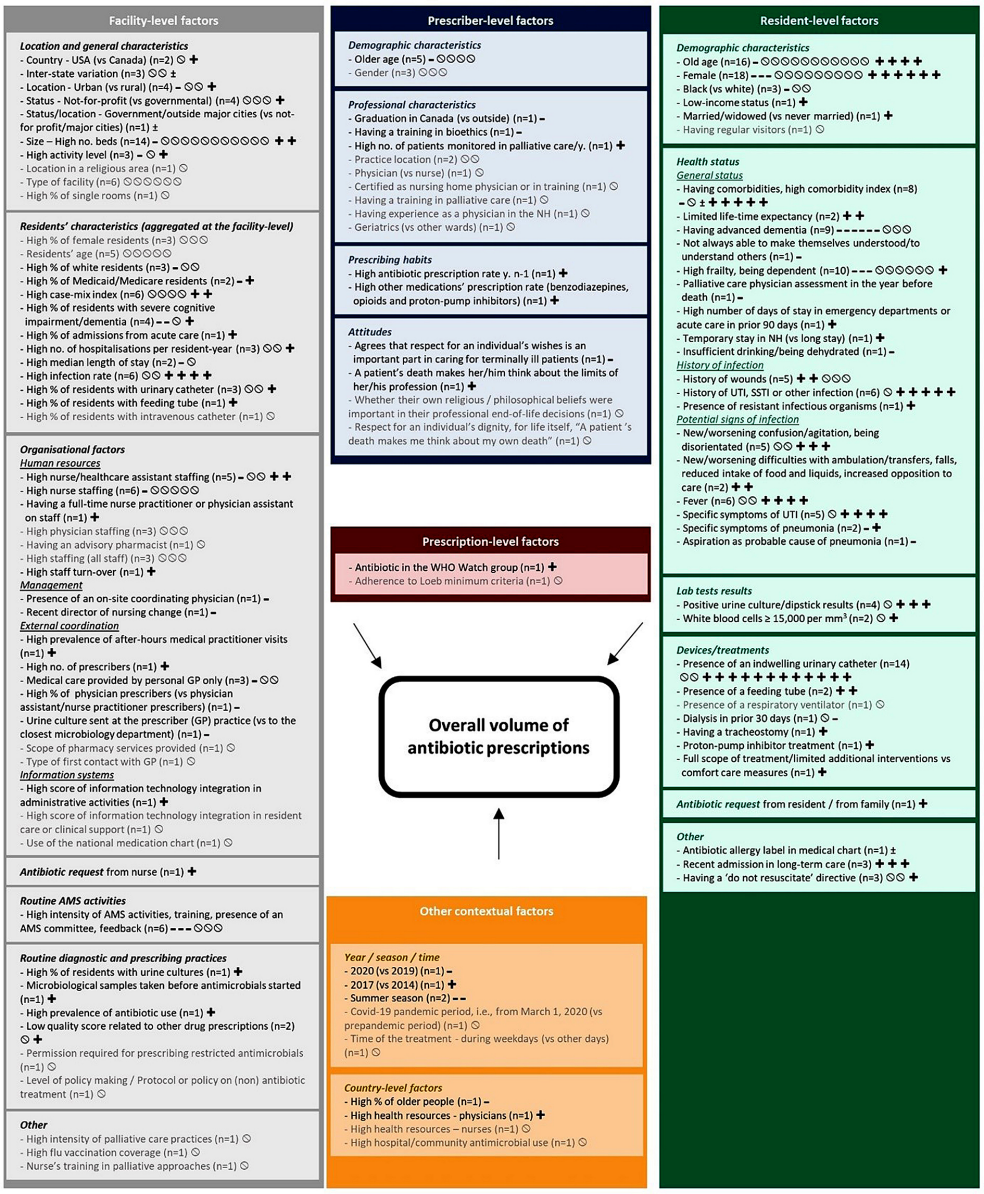
Factors associated with the overall volume of antibiotic prescriptions in LTCFs: summary of the evidence from quantitative studies included in the systematic review (*n* = 47) AMS, antimicrobial stewardship; GP, general practitioner; LTCF, long-term care facility; NH, nursing home; SSTI, skin and soft tissue infection; USA, United States of America; UTI, urinary tract infection; WHO, World Health Organisation. n in brackets: number of studies that investigated this factor. For each study: ∅ Non significant association between the factor investigated and the outcome. **+ ** Significant (*p*≤0.05) positive association (i.e., associated with higher overall volume of antibiotic prescriptions). - Significant (*p*≤0.05) negative association (i.e., associated with lower overall volume of antibiotic prescriptions). **±** Significant positive or negative association, depending on the outcome/factor variable.

Figure 3 displays a summary of the evidence on the factors associated with overall volume of antibiotic prescriptions (see Table S7 for detailed results by study). Out of 47 studies including at least one “overall volume of antibiotic prescriptions” outcome, 43 found at least one statistically significant association with at least one of the factors investigated (see Table S8 for detailed results on these associations and Table S9 for details on the Hill’s criteria for causation).

#### Example

at the facility-level, two studies assessed the association between the LTCF country (USA or Canada) and the overall volume of antibiotic prescriptions; one found no significant association; one found higher antibiotic prescriptions in the USA as compared to Canada.

Factor written (i) in grey: factor investigated in at least one study but which has not been statistically significantly associated with the outcome in any of them; (ii) in black: factor investigated in at least one study and which was statistically significantly associated with the outcome in at least one of them.

#### Resident-level factors

Resident-level factors investigated related to residents’ demographic characteristics (*n* = 18/47), health status, either general status or history/potential signs of infection (*n* = 20/47), lab test results (*n* = 5/47), devices or treatments (*n* = 15/47), or other factors (*n* = 7/47).

Regarding residents’ demographic characteristics, we found mixed results on the association between age, gender, or ethnicity, and overall volume of antibiotic prescriptions.

Regarding the general health status, there was a trend toward higher antibiotic prescriptions among residents having comorbidities (*n* = 5/8) and lower antibiotic prescriptions among residents with dementia (*n* = 6/9). Evidence for both characteristics came from longitudinal studies, adjusted analyses, and showed a dose-response relationship (Table S9). Results also showed a trend toward higher antibiotic prescriptions among residents with a history of infection (*n* = 5/6) and those with potential signs of infection (e.g., confusion/agitation, fever, specific symptoms of urinary tract infection [UTI]).

We also found a trend toward higher antibiotic prescriptions among residents with positive urine culture/dipstick results (*n* = 3/4) and those with an indwelling urinary catheter (*n* = 12/14), who were two to three times more likely to be prescribed an antibiotic (Table S9).

One study found higher antibiotic use in case of antibiotic request by the resident or his/her family (*n* = 1/1).

#### Prescriber-level and prescription-level factors

Prescriber-level factors investigated related to prescribers’ demographic (*n* = 5/47), professional characteristics (*n* = 4/47), prescribing habits (*n* = 2/47), and attitudes (*n* = 1/47).

Few studies (*n* = 1/1 each time) found characteristics associated with lower antibiotic prescriptions (e.g., graduated in Canada, having a training in bioethics) or higher antibiotic prescriptions (e.g., having a high prescription rate for antibiotics or for other medications in the previous year).

#### Facility-level factors

Facility-level factors investigated related to facility’s general characteristics (*n* = 17/47), residents’ characteristics aggregated at the facility-level (*n* = 13/47), organisational factors (*n* = 16/47), routine AMS activities (*n* = 6/47), diagnostic and prescribing practices (*n* = 6/47), and other factors (*n* = 4/47).

Results regarding the association between overall volume of antibiotic prescriptions and LTCF’s general characteristics (e.g., location) were mixed.

Regarding residents’ characteristics aggregated at the facility-level, we found a trend toward higher antibiotic prescriptions in LTCFs with a high infection rate (*n* = 4/6).

As part of the LTCF’s organisational factors, studies showed mixed results regarding the association between the overall volume of antibiotic prescriptions and both healthcare assistant staff and nurse staff. One cross-sectional study found a positive association between staff turnover and volume of antibiotic prescriptions for respiratory tract infections (RTIs) (*n* = 1/1). In terms of management, one cross-sectional study found lower antibiotic prescriptions in LTCFs having an internal coordinating physician (*n* = 1/1). Longitudinal studies also found higher antibiotic prescriptions among LTCFs with a high prevalence of after-hours medical practitioner visits (*n* = 1/1) and a high number of prescribers (*n* = 1/1).

Few studies (*n* = 1/1 each time) found a higher volume of antibiotic prescriptions in case of antibiotic request by nurses or use of some routine diagnostic procedures (e.g., microbiological sample taken before any antibiotic prescription).

#### Other contextual factors

Few studies reported temporal variation in the volume of antibiotic prescriptions. Higher antibiotic prescriptions in 2017 vs. 2014 (*n* = 1/1), lower antibiotic prescriptions in 2020 vs. 2019 (*n* = 1/1) and in summer season (*n* = 2/2) were noted.

### Factors associated with inappropriate antibiotic prescription

**Fig. 4:**
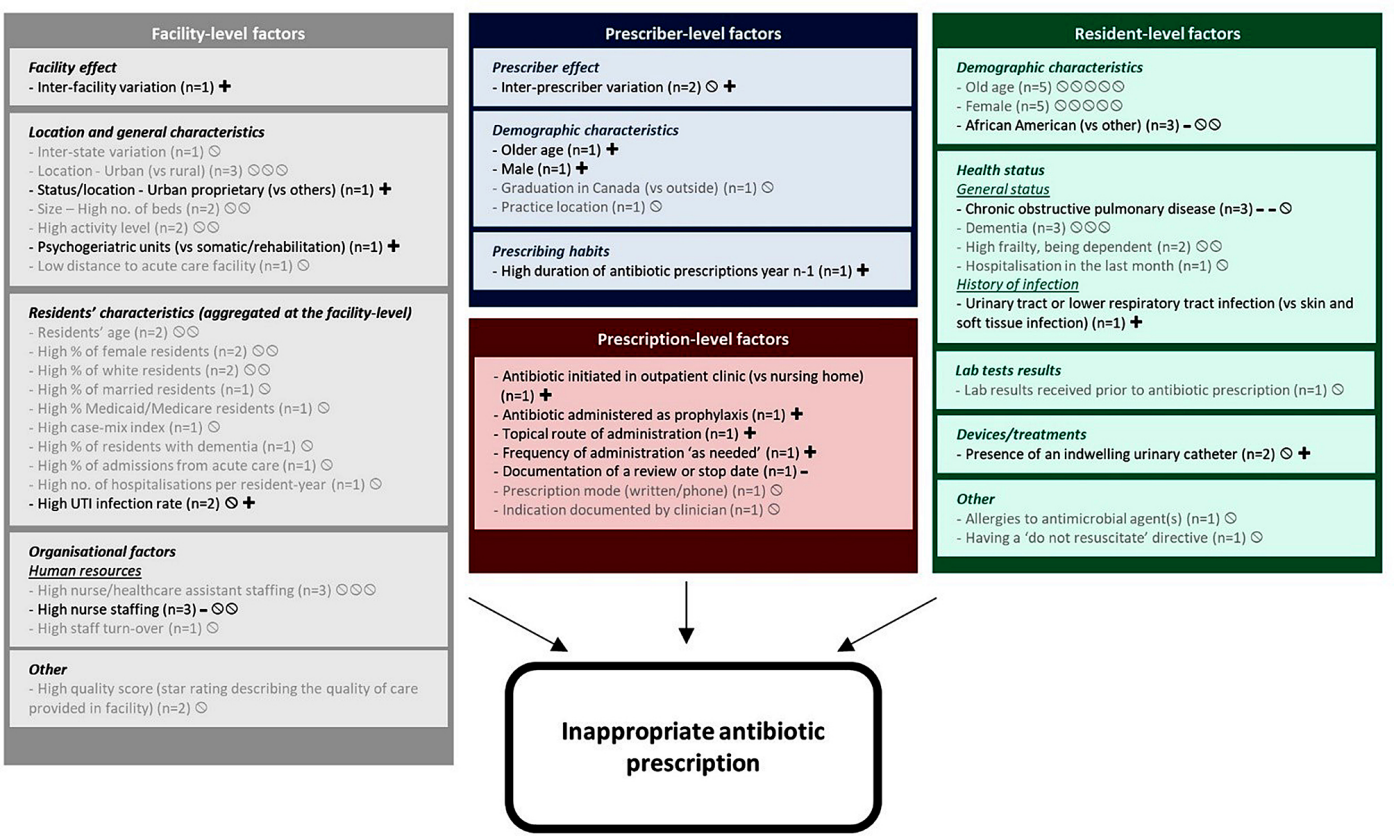
Factors associated with inappropriate antibiotic prescription in LTCFs: summary of the evidence from quantitative studies included in the systematic review (*n* = 12) LTCF, long-term care facility; UTI, urinary tract infection.n in brackets: number of studies that investigated this factor. For each study: ∅ Non significant association between the factor and the outcome. **+ ** Significant (*p*≤0.05) positive association (i.e., associated with a higher risk of inappropriateness of antibiotic prescription). - Significant (*p*≤0.05) negative association (i.e., associated with a lower risk of inappropriateness of antibiotic prescription). ± Significant positive or negative association, depending on the outcome/factor variable.

Figure 4 displays a summary of the evidence on the factors associated with inappropriate antibiotic prescription (see Table S10 for detailed results by study). Out of 12 studies including at least one “inappropriate antibiotic prescription” outcome, 11 found at least one statistically significant association with at least one of the factors investigated (see Table S11 for detailed results on these associations and Table S12 for details on the Hill’s criteria for causation).

#### Example

at the resident-level, three studies assessed the association between ethnicity and inappropriateness of antibiotic prescription; one found that being African American was associated with a lower risk of inappropriate antibiotic prescription; two others found no significant association.

Factor written (i) in grey: factor investigated in at least one study but which has not been statistically significantly associated with the outcome in any of them; (ii) in black: factor investigated in at least one study and which was statistically significantly associated with the outcome in at least one of them.

### Resident-level factors

Resident-level factors investigated related to residents’ demographic characteristics (*n* = 5/12), health status (*n* = 6/12), lab test results (*n* = 1/12), devices or treatments (*n* = 2/12), or other factors (*n* = 2/12).

We found a trend toward a lower risk of inappropriate antibiotic prescription among residents with a diagnosis of chronic obstructive pulmonary disease (*n* = 2/3) and a higher risk among those with UTI or lower RTI (vs. skin and soft tissue infection) (*n* = 1/1).

### Prescriber-level and prescription-level factors

One longitudinal study using adjusted analyses found that older male prescribers were more prone to inappropriate antibiotic prescription than others (*n* = 1/1), as those used to prescribe longer antibiotic treatment duration during the previous year (*n* = 1/1), with a dose-response relationship (Table S12).

Inappropriate antibiotic prescription was more frequent when antibiotic was initiated in an outpatient clinic (vs. in nursing home) (*n* = 1/1), administered as prophylaxis (*n* = 1/1), or topically (*n* = 1/1). On the contrary, documentation of a treatment review or a stop date reduced the risk of inappropriate antibiotic prescription (*n* = 1/1).

### Facility-level factors

One study found significant variation in inappropriate antibiotic prescription between LTCFs (*n* = 1/1) and a higher risk of inappropriate antibiotic prescription in urban proprietary facilities (*n* = 1/1).

## Discussion

### Key results: an interplay of individual, organisational and other contextual factors associated with antibiotic use in LTCFs

In this systematic review of 57 quantitative studies, resident-level factors associated with a higher volume of antibiotic prescriptions included comorbidities, history of infection, potential signs of infection, positive urine culture/dipstick results and indwelling urinary catheter. At the facility-level, the volume of antibiotic prescriptions was positively associated with infection rate. A few studies also showed higher antibiotic prescribing in LTCFs with high staff turnover, prevalence of after-hours medical practitioner visits, and number of prescribers; it was lower in LTCFs having an internal coordinating physician. At the prescriber-level, a few studies found higher antibiotic prescribing among prescribers with a high prescription rate for antibiotics or for other medications in the previous year. A few studies showed that inappropriate antibiotic prescription was more frequent when antibiotic was administered as prophylaxis, or topically; and less frequent when a treatment review or a stop date was documented. Considered as a whole, all these factors might result in a complex conceptual framework underpinning antibiotic use in LCTFs. This framework includes not only individual, but also organisational and other contextual factors associated with antibiotic use.

### Improving infection prevention, and diagnosis process: critical steps to curb antibiotic use in LTCFs

We found that residents with a history of infection were about twice as likely to receive an antibiotic vs. residents with no such history and that this risk was about three times higher among residents with a urinary catheter, a major risk factor for UTI [78]. Evidence derived from our review points at improving infection prevention and control (IPC) as a critical step to reduce antibiotic prescribing in LTCFs. Evidence from the literature suggests that IPC programmes including a multi-modal strategy with four or more WHO core elements were effective in reducing respiratory or MDRO infections, and improving adherence to handwashing practices in LTCF [79].

Results of our systematic review also suggest a key role of the diagnostic process in reducing antibiotic prescribing [30]. Improving the diagnostic process is indeed an essential component of AMS [80]. Potential signs of infection, and positive urine culture/dipstick results were associated with higher antibiotic prescribing. Diagnosis of infections and decision-making about their management in LTCFs are complex and involve several professionals (notably nurses) whose attitudes and practices vary according to the context (e.g., pharmacy accessibility) [81, 82]. A recent review of decision tools used in LTCFs to improve UTI diagnosis showed no consensus as to the clinical criteria on which these tools rely [83]. Besides, living in a high urine-culturing LTCF was independently associated with in an increased likelihood of receiving an antibiotic [30]. Given the high prevalence of asymptomatic bacteriuria in LTCFs, a high proportion of suspected UTIs in LTCFs are indeed not UTIs (estimated at 88% in Latour et al. [84]) and this greatly contributes to unnecessary antibiotic use. Despite some AMS interventions improved UTI diagnosis in LCTFs, their feasibility and sustainability beyond experimental conditions remain unknown, and might be influenced by nursing staff commitment and motivation [83].

### Organisational changes in LTCFs to enhance effective antibiotic stewardship and prescribing practices

Regarding LTCFs’ characteristics, we did not find any effect of facility size or nurse/nursing assistant staff. However, the few studies that investigated other aspects of human resources (e.g., staff turnover), management (e.g., presence of an internal coordinating physician) or organisational features (e.g., prevalence of after-hours medical practitioner visits) showed significant associations with antibiotic prescribing. Staff turnover may impair adoption of AMS strategies and reduce their effect on antibiotic use [10, 85]. On the contrary, the presence of an on-site coordinating physician may facilitate the implementation of AMS strategies by providing extra support and reassurance for the nursing staff [13], in line with the accountability CDC core element of antibiotic stewardship for nursing homes [86]. After-hours medical practitioner visits in LTCFs may enhance antibiotic initiation for reasons including limited knowledge of the resident medical history, limited access to medical records, poor resident current health status, and family or LTCF staff pressure [87]. The majority of current AMS programmes in LTCFs include education strategies and promotion of clinical practice guidelines; a smaller number include audit and feedback interventions and the provision of advice by an infectious disease team [9, 10]. Results from our review suggest that organisational changes requiring more structural adjustments and resource investments may contribute to further reduce antibiotic prescribing in LTCFs. These challenging adjustments may need to limit staff turnover rates, ensure the presence of a professional accountable for the AMS activities, and enhance collaboration between LTCFs and external prescribers, especially physicians engaged in after-hours medical visits (e.g., facilitate access to resident medical records).

Finally, one study included in our review suggested that antibiotic request from resident/family increased the odds of being prescribed an antibiotic by more than 60%. Yet, current LTCF AMS programmes usually target both nurses and physicians but fewer also target residents and/or their family [9, 10]. Inspiration may be drawn from AMS strategies in primary care, where the importance of patients’ expectations is well recognised [88, 89].

### Strengths and limitations

This review has several strengths. As far as we know, this is the first systematic review summarising quantitative evidence about the factors associated with antibiotic use in LTCFs, and highlighting the complexity of the conceptual framework (i.e., including individual, contextual and organisational factors) underpinning antibiotic use in LTCFs. In addition to previous synthesis of qualitative studies [13], our review identified original characteristics that were significantly associated with antibiotic use (both quantity and quality metrics), and provided insight into strengths of associations. Besides, we attempted to provide an assessment of causality based on Hill criteria. It resulted in a conceptual framework underpinning antibiotic use in LTCFs that might help for the development of specific AMS programmes fit to LTCFs settings and suggests the urgent need of institutional changes (e.g., limiting LTCF staff turn-over or hiring on-site physicians) to foster their implementation.

The main limitation is the heterogeneity pertaining to selected outcomes, metrics of the factors investigated, and the methods used across studies that prevented us from performing a meta-analysis. Studies also came from several countries with different LTCF organisation and methods of care delivery that may influence the type of factors associated with antibiotic prescriptions. For instance, request from family may have a lower impact in countries having permanent on-site physicians in LTCFs, and thus many more opportunities for families and physicians to discuss [48]. Another limitation is the small number of studies we could analyses for some factors associated with antibiotic prescribing. For more than half of them, results relied on only one study, hampering any attempt to assess reproducibility (Tables S9 and S12), and highlighting the need for further validation of our conceptual framework.

## Conclusion

Improving infection prevention and control, and diagnostic practices as part of AMS programmes remain critical steps to reduce antibiotic prescribing in LTCFs. Institutional changes to limit staff turnover, ensure the presence of a professional accountable for the AMS activities, and enhance collaboration between LTCFs and external prescribers may also contribute to reduce antibiotic prescribing. The results of this review suggest that reducing antibiotic prescribing in LTCFs requires actions at various levels and the involvement of different stakeholders. Some actions could be implemented at the facility-level, with support from the director, as for example providing staff with tools and training to help them improve infectious diseases diagnosis practices. To improve their collaboration with the external structures that are critical to AMS (e.g., general practitioner private practices, community pharmacies, hospital departments involved in elderly care), LTCFs could also specify in their agreements setting the collaboration with such structures, which stakeholder is in charge of what concerning AMS. Other actions could pertain to other organisational levels external to the LTCF setting, with support from regional and/or national decision makers. These actions could include setting up and funding specialised structures dedicated to AMS to support LTCF in improving their practices. This could also include more general actions targeting the organisation and the resources allocated to LTCFs (e.g., revising salary policies and working conditions to limit staff turnover).

### Electronic supplementary material

Below is the link to the electronic supplementary material.

Supplementary Material 1

Supplementary Material 2

Supplementary Material 3

## Data Availability

All data generated or analysed during this study are included in this published article and its supplementary information files.
