# Supplementary material for: Resident-, prescriber-, and facility-level factors associated with antibiotic use in long-term care facilities: a systematic review of quantitative studies
Source: Antimicrob Resist Infect Control. 2024 Mar 6;13:29. doi: 10.1186/s13756-024-01385-6 (PMC10918961; doi:10.1186/s13756-024-01385-6)
Supplement: Supplementary file 1 — Supplementary Material 1 [file 13756_2024_1385_MOESM1_ESM.docx]

**Resident-, Prescriber-, and Facility-level Factors Associated with Antibiotic Use in Long-Term Care Facilities: A Systematic Review of Quantitative Studies**

**Supplementary data**

**Table S1** PRISMA 2020 Checklist

| **Section and Topic** | **Item #** | **Checklist item** | **Location where item is reported** |
| --- | --- | --- | --- |
| **TITLE** | | |  |
| Title | 1 | Identify the report as a systematic review. | Title, p.1 |
| **ABSTRACT** | | |  |
| Abstract | 2 | See the PRISMA 2020 for Abstracts checklist. | Abstract, p.2 |
| **INTRODUCTION** | | |  |
| Rationale | 3 | Describe the rationale for the review in the context of existing knowledge. | Introduction, p.4-5 |
| Objectives | 4 | Provide an explicit statement of the objective(s) or question(s) the review addresses. | Introduction, last §, p.5 |
| **METHODS** | | |  |
| Eligibility criteria | 5 | Specify the inclusion and exclusion criteria for the review and how studies were grouped for the syntheses. | Methods, § Eligibility criteria, p.6 |
| Information sources | 6 | Specify all databases, registers, websites, organisations, reference lists and other sources searched or consulted to identify studies. Specify the date when each source was last searched or consulted. | Methods, § Search strategy, p.5 |
| Search strategy | 7 | Present the full search strategies for all databases, registers and websites, including any filters and limits used. | Methods, § Search strategy, p.5 and Table S2 |
| Selection process | 8 | Specify the methods used to decide whether a study met the inclusion criteria of the review, including how many reviewers screened each record and each report retrieved, whether they worked independently, and if applicable, details of automation tools used in the process. | Methods, § Eligibility criteria, p.6 |
| Data collection process | 9 | Specify the methods used to collect data from reports, including how many reviewers collected data from each report, whether they worked independently, any processes for obtaining or confirming data from study investigators, and if applicable, details of automation tools used in the process. | Methods, § Data extraction, p.6-7 |
| Data items | 10a | List and define all outcomes for which data were sought. Specify whether all results that were compatible with each outcome domain in each study were sought (e.g. for all measures, time points, analyses), and if not, the methods used to decide which results to collect. | Methods, § Eligibility criteria, p.6 |
|  | 10b | List and define all other variables for which data were sought (e.g. participant and intervention characteristics, funding sources). Describe any assumptions made about any missing or unclear information. | Methods, § Data extraction, p.7 |
| Study risk of bias assessment | 11 | Specify the methods used to assess risk of bias in the included studies, including details of the tool(s) used, how many reviewers assessed each study and whether they worked independently, and if applicable, details of automation tools used in the process. | Methods, § Quality assessment, p.7 |
| Effect measures | 12 | Specify for each outcome the effect measure(s) (e.g. risk ratio, mean difference) used in the synthesis or presentation of results. | Methods, § Data extraction, p.7 |
| Synthesis methods | 13a | Describe the processes used to decide which studies were eligible for each synthesis (e.g. tabulating the study intervention characteristics and comparing against the planned groups for each synthesis (item #5)). | Methods, § Eligibility criteria, p.6 |
|  | 13b | Describe any methods required to prepare the data for presentation or synthesis, such as handling of missing summary statistics, or data conversions. | N/A |
|  | 13c | Describe any methods used to tabulate or visually display results of individual studies and syntheses. | N/A |
|  | 13d | Describe any methods used to synthesize results and provide a rationale for the choice(s). If meta-analysis was performed, describe the model(s), method(s) to identify the presence and extent of statistical heterogeneity, and software package(s) used. | Methods, § Data synthesis, p.7-8 |
|  | 13e | Describe any methods used to explore possible causes of heterogeneity among study results (e.g. subgroup analysis, meta-regression). | N/A |
|  | 13f | Describe any sensitivity analyses conducted to assess robustness of the synthesized results. | N/A |
| Reporting bias assessment | 14 | Describe any methods used to assess risk of bias due to missing results in a synthesis (arising from reporting biases). | N/A |
| Certainty assessment | 15 | Describe any methods used to assess certainty (or confidence) in the body of evidence for an outcome. | Methods, § Data synthesis, p.8 and Tables S8 and S11 |
| **RESULTS** | | |  |
| Study selection | 16a | Describe the results of the search and selection process, from the number of records identified in the search to the number of studies included in the review, ideally using a flow diagram. | Results, § Study selection and characteristics, p.9 and Figure 1 |
|  | 16b | Cite studies that might appear to meet the inclusion criteria, but which were excluded, and explain why they were excluded. | Figure 1 and Table S4 |
| Study characteristics | 17 | Cite each included study and present its characteristics. | Results, § Study selection and characteristics and Figure 2 |
| Risk of bias in studies | 18 | Present assessments of risk of bias for each included study. | Table S3 |
| Results of individual studies | 19 | For all outcomes, present, for each study: (a) summary statistics for each group (where appropriate) and (b) an effect estimate and its precision (e.g. confidence/credible interval), ideally using structured tables or plots. | Tables S6-S7 and Table S9-S10 |
| Results of syntheses | 20a | For each synthesis, briefly summarise the characteristics and risk of bias among contributing studies. | Results, § Factors associated with overall volume of antibiotic prescriptions and § Factors associated with inappropriate antibiotic prescription |
|  | 20b | Present results of all statistical syntheses conducted. If meta-analysis was done, present for each the summary estimate and its precision (e.g. confidence/credible interval) and measures of statistical heterogeneity. If comparing groups, describe the direction of the effect. | N/A |
|  | 20c | Present results of all investigations of possible causes of heterogeneity among study results. | N/A |
|  | 20d | Present results of all sensitivity analyses conducted to assess the robustness of the synthesized results. | N/A |
| Reporting biases | 21 | Present assessments of risk of bias due to missing results (arising from reporting biases) for each synthesis assessed. | N/A |
| Certainty of evidence | 22 | Present assessments of certainty (or confidence) in the body of evidence for each outcome assessed. | Tables S8 and S11 |
| **DISCUSSION** | | |  |
| Discussion | 23a | Provide a general interpretation of the results in the context of other evidence. | Discussion, p.13-15 |
|  | 23b | Discuss any limitations of the evidence included in the review. | Discussion, § Strengths and limitations, p.15-16 |
|  | 23c | Discuss any limitations of the review processes used. | Discussion, § Strengths and limitations, p.16 |
|  | 23d | Discuss implications of the results for practice, policy, and future research. | Discussion, p.15-16 |
| **OTHER INFORMATION** | | |  |
| Registration and protocol | 24a | Provide registration information for the review, including register name and registration number, or state that the review was not registered. | Methods, § Search strategy, p.5 |
|  | 24b | Indicate where the review protocol can be accessed, or state that a protocol was not prepared. | Methods, § Search strategy, p.5 |
|  | 24c | Describe and explain any amendments to information provided at registration or in the protocol. | N/A |
| Support | 25 | Describe sources of financial or non-financial support for the review, and the role of the funders or sponsors in the review. | § Funding, p.17 |
| Competing interests | 26 | Declare any competing interests of review authors. | § Conflict of interest disclosure, p.17 |
| Availability of data, code and other materials | 27 | Report which of the following are publicly available and where they can be found: template data collection forms; data extracted from included studies; data used for all analyses; analytic code; any other materials used in the review. | Data used for all analyses: Tables S6-S11 |

*From:*  Page MJ, McKenzie JE, Bossuyt PM, Boutron I, Hoffmann TC, Mulrow CD, et al. The PRISMA 2020 statement: an updated guideline for reporting systematic reviews. BMJ 2021;372:n71. doi: 10.1136/bmj.n71

For more information, visit: <http://www.prisma-statement.org/>

**Table S2** Search strategies

| **MEDLINE (Pubmed) search strategy on the 24^th^ May 2022** | | |
| --- | --- | --- |
| **Sequence** | **Query** | **No. records** |
| #1 Antibiotic | antibiotic*[Title/Abstract] OR anti-biotic*[Title/Abstract] OR antibacterial[Title/Abstract] OR anti-bacterial[Title/Abstract] OR antimicrobi*[Title/Abstract] OR anti-microbi*[Title/Abstract] OR antiinfective[Title/Abstract] OR anti-infective [Title/Abstract] OR Anti-Bacterial Agents[MeSH Terms] OR Anti-Infective Agents[Mesh Terms] | 1,095,327 |
| #2 *Nursing home_1* | long-term care[Title/Abstract] OR long term care[Title/Abstract] OR residential [Title/Abstract] OR Veterans [Title/Abstract] OR aged care[Title/Abstract] OR assisted living[Title/Abstract] | 103,671 |
| #3 *Nursing home_2* | facilit*[Title/Abstract] OR center*[Title/Abstract] OR centre*[Title/Abstract] OR unit*[Title/Abstract] OR hospital*[Title/Abstract] | 3,851,381 |
| #4 *Nursing home_3* | #2 AND #3 | 48,197 |
| #5 *Nursing home_4* | Nursing Home*[Title/Abstract] OR Home* for the aged [Title/Abstract] OR LTCF[Title/Abstract] OR homes for the aged[MeSH Terms] OR assisted living facilities[MeSH Terms] OR nursing homes[MeSH Terms] OR Residential Facilities[Mesh Terms] OR Long-Term Care [Mesh Terms] | 89,400 |
| #6 *Nursing homes_all* | #4 OR #5  ((long-term care[Title/Abstract] OR long term care[Title/Abstract] OR residential [Title/Abstract] OR Veterans [Title/Abstract] OR aged care[Title/Abstract] OR assisted living[Title/Abstract]) AND (facilit*[Title/Abstract] OR center*[Title/Abstract] OR centre*[Title/Abstract] OR unit*[Title/Abstract] OR hospital*[Title/Abstract])) OR (Nursing Home*[Title/Abstract] OR Home* for the aged [Title/Abstract] OR LTCF[Title/Abstract] OR homes for the aged[MeSH Terms] OR assisted living facilities[MeSH Terms] OR nursing homes[MeSH Terms] OR Residential Facilities[Mesh Terms] OR Long-Term Care [Mesh Terms]) | 124,224 |
| #7 GPs | General Practitioners[Mesh Terms] OR Family Practice[Mesh Terms] OR Primary Health Care[Mesh Terms] OR Physicians, Primary Care[Mesh Terms] OR Ambulatory Care[Mesh Terms] OR general practi*[Title/Abstract] OR family practi*[Title/Abstract] OR primary care[Title/Abstract] OR primary healthcare[Title/Abstract] OR family doctor*[Title/Abstract] OR family med*[Title/Abstract] OR ambulatory care[Title/Abstract] | 430,273 |
| #8 Older adults | Aged[MeSH Terms] OR Frail Elderly[MeSH Terms] OR elderl*[Title/Abstract] OR older adult*[Title/Abstract] OR senior*[Title/Abstract] OR geriatric*[Title/Abstract] | 3,549,897 |
| #9 GPs AND Older adults | #7 AND #8 | 102,745 |
| #10 nursing home OR (GPs AND Older adults) | #6 OR #9 | 219,992 |
| #11 Antibiotic AND nursing home OR (GPs AND Older adults)) | #1 AND #10 | 7,417 |
| #12 Associated factors | characteristic*[Title/Abstract] OR factor*[Title/Abstract] OR determinant*[Title/Abstract] OR correlat*[Title/Abstract] OR influence* [Title/Abstract] OR associat* [Title/Abstract] OR Epidemiologic Factors[Mesh Terms] OR Practice patterns, physician [Mesh Terms] | 11,392,049 |
| #13 final_1 | #11 AND #12 | 4,318 |
| #14 *Language* | (English[Language]) OR (French[Language]) OR (Turkish [language]) | 30,097,518 |
| #15 final_2 | #13 AND #14 | 4;061 |
| **MEDLINE (Pubmed) complementary search strategy on the 9th February 2024** | | |
| #1 Antibiotic | antibiotic*[Title/Abstract] OR anti-biotic*[Title/Abstract] OR antibacterial[Title/Abstract] OR anti-bacterial[Title/Abstract] OR antimicrobi*[Title/Abstract] OR anti-microbi*[Title/Abstract] OR antiinfective[Title/Abstract] OR anti-infective [Title/Abstract] OR Anti-Bacterial Agents[MeSH Terms] OR Anti-Infective Agents[Mesh Terms] | 1,201,550 |
| #2 *Nursing home_1* | long-term care[Title/Abstract] OR long term care[Title/Abstract] OR residential [Title/Abstract] OR Veterans [Title/Abstract] OR aged care[Title/Abstract] OR assisted living[Title/Abstract] | 117,331 |
| #3 *Nursing home_2* | facilit*[Title/Abstract] OR center*[Title/Abstract] OR centre*[Title/Abstract] OR unit*[Title/Abstract] OR hospital*[Title/Abstract] | 4,357,359 |
| #4 *Nursing home_3* | #2 AND #3 | 54,695 |
| #5 *Nursing home_4* | Nursing Home*[Title/Abstract] OR Home* for the aged [Title/Abstract] OR LTCF[Title/Abstract] OR homes for the aged[MeSH Terms] OR assisted living facilities[MeSH Terms] OR nursing homes[MeSH Terms] OR Residential Facilities[Mesh Terms] OR Long-Term Care [Mesh Terms] | 93,788 |
| #6 *Nursing homes_all* | #4 OR #5  ((long-term care[Title/Abstract] OR long term care[Title/Abstract] OR residential [Title/Abstract] OR Veterans [Title/Abstract] OR aged care[Title/Abstract] OR assisted living[Title/Abstract]) AND (facilit*[Title/Abstract] OR center*[Title/Abstract] OR centre*[Title/Abstract] OR unit*[Title/Abstract] OR hospital*[Title/Abstract])) OR (Nursing Home*[Title/Abstract] OR Home* for the aged [Title/Abstract] OR LTCF[Title/Abstract] OR homes for the aged[MeSH Terms] OR assisted living facilities[MeSH Terms] OR nursing homes[MeSH Terms] OR Residential Facilities[Mesh Terms] OR Long-Term Care [Mesh Terms]) | 133,735 |
| #7 GPs | General Practitioners[Mesh Terms] OR Family Practice[Mesh Terms] OR Primary Health Care[Mesh Terms] OR Physicians, Primary Care[Mesh Terms] OR Ambulatory Care[Mesh Terms] OR general practi*[Title/Abstract] OR family practi*[Title/Abstract] OR primary care[Title/Abstract] OR primary healthcare[Title/Abstract] OR family doctor*[Title/Abstract] OR family med*[Title/Abstract] OR ambulatory care[Title/Abstract] | 462,362 |
| #8 Older adults | Aged[MeSH Terms] OR Frail Elderly[MeSH Terms] OR elderl*[Title/Abstract] OR older adult*[Title/Abstract] OR senior*[Title/Abstract] OR geriatric*[Title/Abstract] | 3,656,474 |
| #9 GPs AND Older adults | #7 AND #8 | 106,926 |
| #10 nursing home OR (GPs AND Older adults) | #6 OR #9 | 233,293 |
| #11 Antibiotic AND nursing home OR (GPs AND Older adults)) | #1 AND #10 | 7,847 |
| #12 Associated factors | characteristic*[Title/Abstract] OR factor*[Title/Abstract] OR determinant*[Title/Abstract] OR correlat*[Title/Abstract] OR influence* [Title/Abstract] OR associat* [Title/Abstract] OR Epidemiologic Factors[Mesh Terms] OR Practice patterns, physician [Mesh Terms] | 12,630,436 |
| #13 final_1 | #11 AND #12 | 4,596 |
| #14 English | English[Language] | 32,034,382 |
| #15 French | French[Language] | 769,860 |
| #16 Turkish | Turkish [Language] | 11,384 |
| #17 final_2 | #13 final_1 NOT #14 NOT #15 NOT #16 | 262 |
| #18 publication date | ("1000/01/01"[Date - Publication] : "2022/05/24"[Date - Publication]) | 34,408,397 |
| #19 final_3 | #17 AND #18 | 258 |
| **Cochrane Library search strategy on the 24^th^ May 2022** | | |
|  | (antibiotic* OR anti-biotic* OR antibacterial OR anti-bacterial OR antimicrobi* OR anti-microbi* OR antiinfective OR anti-infective):ti,ab,kw OR MesH descriptor: [Anti-Bacterial Agents] explode all trees OR MesH descriptor: [Anti-Infective Agents] explode all trees  AND  (((long-term care OR long term care OR residential OR Veterans OR aged care OR assisted living): ti,ab,kw AND (facilit* OR center* OR centre* OR unit* OR hospital*)ti,ab,kw) OR (Nursing home* OR home* for the aged OR LTCF):ti,ab,kw OR MesH descriptor: [homes for the aged] explode all trees OR MesH descriptor: [Assisted living facilities] explode all trees OR MesH descriptor: [Nursing homes] explode all trees OR MesH descriptor: [residential facilities] explode all trees OR MesH descriptor: [Long-term Care] explode all trees)  OR  ((MesH descriptor: [Genral Practitioners] explode all trees OR MesH descriptor: [Family practice] explode all trees OR MesH descriptor: [Primary Health care] explode all trees OR MesH descriptor: [Physician, Primary Care] explode all trees OR MesH descriptor: [Ambulatory care] explode all trees OR (general practi* OR family practi* OR primary care OR primary healthcare OR family doctor OR family med* OR ambulatory care):ti,ab,kw) AND (MesH descriptor: [Aged] explode all trees OR MesH descriptor: [Frail Elderly] explode all trees OR (elderl* OR older adul* OR senior* OR geriatric*):ti,ab,kw)))  AND  (characteristic* OR factor* OR determinent* OR correlat* OR influence* OR associat*):ti,ab,kw OR MesH descriptor: [Epidemiologic Factors] explode all trees OR MesH descriptor: [Practice patterns, physician] explode all trees | 2,404 |
| **Embase search strategy on the 24^th^ May 2022** | | |
|  | (. AND &apos;antibiotic*&apos;:ab,ti OR &apos;anti biotic*&apos;:ab,ti OR antibacterial:ab,ti OR &apos;anti bacterial&apos;:ab,ti OR &apos;antimicrobi*&apos;:ab,ti OR &apos;anti microbi*&apos;:ab,ti OR &apos;antiinfective&apos;:ab,ti OR &apos;anti infective&apos;:ab,ti) AND (. AND (&apos;long-term care&apos;:ab,ti OR &apos;long term care&apos;:ab,ti OR &apos;residential&apos;:ab,ti OR &apos;veterans&apos;:ab,ti OR &apos;aged care&apos;:ab,ti) AND (&apos;facilit*&apos;:ab,ti OR &apos;center*&apos;:ab,ti OR &apos;centre*&apos;:ab,ti OR &apos;unit*&apos;:ab,ti OR &apos;hospital*&apos;:ab,ti) OR &apos;nursing home*&apos;:ab,ti OR &apos;home* for the aged&apos;:ab,ti OR &apos;ltcf&apos;:ab,ti OR ((&apos;general practi*&apos;:ab,ti OR &apos;family practi*&apos;:ab,ti OR &apos;primary care&apos;:ab,ti OR &apos;primary healthcare&apos;:ab,ti OR &apos;family doctor*&apos;:ab,ti OR &apos;family med*&apos;:ab,ti OR &apos;ambulatory care&apos;:ab,ti) AND (&apos;elderl*&apos;:ab,ti OR &apos;older adult*&apos;:ab,ti OR &apos;senior*&apos;:ab,ti OR &apos;geriatric*&apos;:ab,ti))) AND (. AND &apos;characteristic*&apos;:ab,ti OR &apos;factor*&apos;:ab,ti OR &apos;determinent*&apos;:ab,ti OR &apos;correlat*&apos;:ab,ti OR &apos;influence*&apos;:ab,ti OR &apos;associat*&apos;:ab,ti) | 2,804 |
| **PsycInfo search strategy on the 24^th^ May 2022** | | |
|  | TI ( antibiotic* OR anti-biotic* OR antibacterial OR anti-bacterial OR antimicrobi* OR anti-microbi* OR antiinfective OR anti-infective ) OR AB ( antibiotic* OR anti-biotic* OR antibacterial OR anti-bacterial OR antimicrobi* OR anti-microbi* OR antiinfective OR anti-infective )  AND  TI (( long-term care OR long term care OR residential O veterans OR aged care ) AND TI ( faciliti* OR center* OR centre* OR unit* OR hospital* )) OR AB (( long-term care OR long term care OR residential O veterans OR aged care ) AND AB ( faciliti* OR center* OR centre* OR unit* OR hospital* ))  OR  TI ( Nursing home* OR home* for the aged OR LTCF ) OR AB ( Nursing home* OR home* for the aged OR LTCF )  OR  TI (general practi* OR family practi* OR primary care OR primary healthcare OR family doctor* OR family med* OR ambulatory care) OR AB (general practi* OR family practi* OR primary care OR primary healthcare OR family doctor* OR family med* OR ambulatory care)  AND TI (elderl*[Title/Abstract] OR older adult* OR senior* OR geriatric*) OR AB (elderl*[Title/Abstract] OR older adult* OR senior* OR geriatric*)  AND  TI ( characteristic* OR factor* OR determinant* OR correlat* OR influence*OR associat* ) OR AB ( characteristic* OR factor* OR determinant* OR correlat* OR influence*OR associat* ) | 53 |
| **ScienceDirect search strategy on the 24^th^ May 2022** | | |
|  | (Antibiotic OR antiinfective ) AND ((nursing home OR long term care OR home for the aged) OR (general practice OR family practice)) AND (characteristic OR determinant) | 179 |
| **Web of Science search strategy on the 24^th^ May 2022** | | |
|  | (TI=(antibiotic* OR anti-biotic* OR antibacterial* OR anti-bacterial* OR antimicrobi* OR anti-microbi* OR antiinfective OR anti-infective)) OR AB=(antibiotic* OR anti-biotic* OR antibacterial* OR anti-bacterial* OR antimicrobi* OR anti-microbi* OR antiinfective OR anti-infective)  AND  ((AB=(("long-term care" OR "long term care" OR residential OR Veterans OR "aged care" OR "assisted living") AND (facilit* OR center* OR centre* OR unit* OR hospital*)) OR TI=(("long-term care" OR "long term care" OR residential OR Veterans OR "aged care" OR "assisted living") AND (facilit* OR center* OR centre* OR unit* OR hospital*))) OR (AB=("Nursing Home*" OR "Home* for the aged" OR LTCF) OR TI=("Nursing Home*" OR "Home* for the aged" OR LTCF)) OR (TI=("general practi*" OR "family practi*" OR "primary care" OR "primary healthcare" OR "family doctor*" OR "family med*" OR "ambulatory care")) OR AB=("general practi*" OR "family practi*" OR "primary care" OR "primary healthcare" OR "family doctor*" OR "family med*" OR "ambulatory care") AND ((TI=(elderl* OR "older adult*" OR senior* OR geriatric*)) OR AB=(elderl* OR "older adult*" OR senior* OR geriatric*)  AND  AB=(characteristic* OR factor* OR determinant* OR correlat* OR influence* OR associat*) OR TI=(characteristic* OR factor* OR determinant* OR correlat* OR influence* OR associat*) | 1,567 |

**Table S3** Quality of reporting* of the studies included in the systematic review (n=57)

| First author, date of publication | **Design** | **Setting** | | | **Participants** | | | **Variables** | | **Data sources / measurement** | | **Bias**** | **Study size** | **Quantitative variables** | **Statistical methods** | | **Score** |
| --- | --- | --- | --- | --- | --- | --- | --- | --- | --- | --- | --- | --- | --- | --- | --- | --- | --- |
|  | Indicate study design early in the paper | Describe the setting (recruitment sites or sources) | Describe the locations (country, town...) | Describe relevant dates (periods of recruitment, data collection...) | Give the eligibility criteria | Give the sources of selection of participants | Give the methods of selection of participants (simple random, systematic, stratified, cluster) | Clearly define all outcomes | Clearly define all factors investigated | For each outcome, give sources of data and details of methods of assessment | For each factor investigated, give sources of data and details of methods of assessment | Describe any efforts to address potential sources of bias: (1) Selection bias; (2) Measure bias; (3) Confusion bias | Explain how the study size was arrived at (i.e., the flow chart) | Explain how quantitative variables were handled in the analyses | Describe all statistical methods | Explain how missing data were addressed |  |
| Appaneal H.J., 2021 | 1 | 1 | 0 | 1 | 1 | 1 | 1 | 1 | 0 | 1 | 0 | 0 | 0 | 0 | 1 | 0 | 9 |
| Basso I. and Dimonte V., 2013 | 1 | 1 | 1 | 1 | 1 | 1 | 1 | 0 | 1 | 1 | 1 | 0 | 1 | 1 | 1 | 0 | 13 |
| Bennett N., 2018 | 1 | 1 | 1 | 1 | 1 | 1 | 1 | 1 | 1 | 1 | 1 | 0 | 0 | 1 | 1 | 0 | 13 |
| Bennett N.J., 2019 | 1 | 1 | 1 | 1 | 1 | 1 | 1 | 0 | 0 | 1 | 1 | 0 | 0 | 1 | 0 | 0 | 10 |
| Benoit S.R., 2008 | 1 | 1 | 1 | 1 | 0 | 1 | 1 | 1 | 1 | 1 | 1 | 1 | 0 | 1 | 1 | 0 | 13 |
| Boivin Y., 2013 | 1 | 1 | 1 | 1 | 1 | 0 | 1 | 1 | 1 | 1 | 1 | 0 | 0 | 1 | 1 | 0 | 12 |
| Brown K.A., 2020 | 1 | 1 | 1 | 1 | 1 | 1 | 1 | 1 | 1 | 1 | 1 | 1 | 1 | 1 | 1 | 0 | 15 |
| Buehrle D.J., 2020 | 1 | 1 | 1 | 1 | 1 | 0 | 0 | 1 | 1 | 0 | 0 | 0 | 0 | 1 | 1 | 0 | 9 |
| Campitelli M.A., 2021 | 1 | 1 | 1 | 1 | 1 | 1 | 1 | 1 | 1 | 1 | 1 | 0 | 1 | 1 | 1 | 0 | 14 |
| Cohen C.C., 2022 | 1 | 1 | 1 | 1 | 1 | 1 | 1 | 1 | 1 | 1 | 1 | 0 | 1 | 1 | 1 | 0 | 14 |
| Daneman N., 2011 | 1 | 1 | 1 | 1 | 1 | 1 | 1 | 1 | 1 | 1 | 1 | 1 | 0 | 1 | 1 | 0 | 14 |
| Daneman N., 2013 | 1 | 1 | 1 | 1 | 1 | 1 | 1 | 1 | 1 | 1 | 1 | 0 | 0 | 1 | 1 | 0 | 13 |
| Daneman N., 2017 | 1 | 1 | 1 | 1 | 1 | 1 | 1 | 0 | 1 | 1 | 1 | 0 | 1 | 1 | 1 | 0 | 13 |
| Durand M., 2021 | 0 | 1 | 1 | 1 | 1 | 1 | 1 | 0 | 1 | 1 | 1 | 0 | 0 | 1 | 1 | 0 | 11 |
| Gouin K.A., 2022 | 0 | 1 | 1 | 1 | 0 | 1 | 0 | 1 | 1 | 1 | 1 | 0 | 0 | 1 | 1 | 0 | 10 |
| Harbin N.J., 2020 | 1 | 1 | 1 | 1 | 1 | 1 | 1 | 1 | 1 | 1 | 1 | 0 | 0 | 1 | 1 | 0 | 13 |
| Hendricksen M, 2021 | 1 | 1 | 1 | 1 | 1 | 1 | 1 | 1 | 1 | 1 | 1 | 0 | 0 | 1 | 1 | 0 | 13 |
| Hendriks S.A., 2017 | 1 | 1 | 1 | 1 | 1 | 1 | 0 | 0 | 1 | 1 | 1 | 0 | 0 | 1 | 1 | 1 | 12 |
| Héquet D., 2021 | 1 | 1 | 1 | 1 | 1 | 1 | 1 | 1 | 1 | 1 | 1 | 0 | 0 | 1 | 0 | 0 | 12 |
| Hutt E., 2008 | 1 | 1 | 1 | 1 | 1 | 1 | 0 | 0 | 1 | 1 | 1 | 0 | 0 | 1 | 1 | 0 | 11 |
| Kistler C.E., 2017 | 0 | 1 | 1 | 1 | 1 | 1 | 1 | 1 | 0 | 1 | 1 | 0 | 0 | 1 | 0 | 1 | 11 |
| Kistler C.E., 2020 | 1 | 1 | 1 | 1 | 1 | 1 | 0 | 0 | 1 | 1 | 1 | 0 | 1 | 1 | 1 | 0 | 12 |
| Kolodziej L.M., 2022 | 1 | 1 | 1 | 1 | 1 | 0 | 1 | 0 | 0 | 0 | 0 | 0 | 0 | 1 | 1 | 1 | 9 |
| Loeb M., 2004 | 1 | 1 | 1 | 1 | 0 | 0 | 1 | 0 | 1 | 1 | 1 | 0 | 0 | 1 | 1 | 0 | 10 |
| Mayne S., 2018 | 1 | 1 | 1 | 1 | 1 | 1 | 1 | 1 | 1 | 1 | 1 | 1 | 0 | 1 | 1 | 0 | 14 |
| McClean P., 2011 | 1 | 1 | 1 | 1 | 1 | 0 | 1 | 0 | 1 | 0 | 0 | 0 | 1 | 1 | 1 | 0 | 10 |
| Mehr D.R., 2003 | 1 | 1 | 1 | 0 | 1 | 1 | 0 | 1 | 1 | 0 | 1 | 0 | 0 | 1 | 1 | 0 | 10 |
| Montgomery P., 1995 | 1 | 1 | 1 | 1 | 1 | 1 | 1 | 1 | 1 | 1 | 1 | 0 | 0 | 1 | 1 | 0 | 13 |
| Mylotte J.M., 1999 | 1 | 1 | 1 | 1 | 0 | 1 | 1 | 1 | 1 | 1 | 0 | 0 | 0 | 1 | 1 | 0 | 11 |
| Mylotte J.M., 2003 | 1 | 1 | 1 | 1 | 1 | 1 | 1 | 1 | 1 | 0 | 1 | 0 | 0 | 1 | 1 | 0 | 12 |
| Mylotte J.M., 2005 | 1 | 1 | 1 | 1 | 1 | 1 | 1 | 1 | 1 | 0 | 1 | 0 | 0 | 1 | 1 | 0 | 12 |
| Olsho L.E.W., 2013 | 1 | 1 | 1 | 1 | 0 | 1 | 1 | 0 | 0 | 0 | 1 | 0 | 1 | 0 | 0 | 0 | 8 |
| Onder G. 2013 | 0 | 1 | 1 | 0 | 1 | 0 | 1 | 1 | 1 | 1 | 1 | 0 | 1 | 1 | 1 | 1 | 12 |
| Pengo V., 2017 | 0 | 1 | 1 | 1 | 1 | 0 | 1 | 1 | 1 | 1 | 1 | 0 | 0 | 1 | 1 | 0 | 11 |
| Phillips C.D., 2012 | 0 | 1 | 1 | 1 | 0 | 1 | 1 | 1 | 1 | 1 | 1 | 0 | 0 | 1 | 1 | 0 | 11 |
| Pulia M., 2018 | 1 | 1 | 1 | 1 | 1 | 1 | 1 | 1 | 1 | 1 | 1 | 0 | 0 | 0 | 1 | 0 | 12 |
| Quinn K.L., 2019 | 1 | 1 | 1 | 1 | 1 | 1 | 1 | 1 | 1 | 1 | 1 | 0 | 1 | 1 | 1 | 0 | 14 |
| Raban M.Z., 2020 | 1 | 1 | 1 | 1 | 1 | 1 | 1 | 1 | 1 | 1 | 1 | 0 | 1 | 1 | 1 | 0 | 14 |
| Roughead E.E., 2009 | 1 | 1 | 1 | 1 | 1 | 1 | 1 | 0 | 1 | 1 | 1 | 0 | 0 | 0 | 1 | 0 | 11 |
| Roukens M. 2017 | 1 | 1 | 1 | 1 | 1 | 0 | 1 | 1 | 1 | 1 | 1 | 0 | 1 | 1 | 1 | 1 | 14 |
| Rummukainen M.-L., 2013 | 1 | 1 | 1 | 1 | 0 | 0 | 1 | 0 | 0 | 1 | 1 | 0 | 0 | 0 | 0 | 0 | 7 |
| Saxena F.E., 2019 | 1 | 1 | 1 | 1 | 1 | 1 | 1 | 1 | 1 | 1 | 1 | 1 | 0 | 1 | 1 | 0 | 14 |
| Sloane P.D., 2017 | 0 | 1 | 1 | 1 | 1 | 1 | 1 | 1 | 1 | 1 | 1 | 0 | 1 | 1 | 1 | 0 | 13 |
| Sluggett J.K., 2021 | 1 | 1 | 1 | 1 | 1 | 1 | 1 | 1 | 1 | 1 | 1 | 0 | 1 | 1 | 1 | 0 | 14 |
| Smith C.M., 2020 | 1 | 1 | 1 | 1 | 1 | 1 | 1 | 1 | 1 | 1 | 1 | 0 | 1 | 1 | 1 | 0 | 14 |
| Sommer-Larsen S.D, 2021 | 1 | 1 | 1 | 1 | 1 | 1 | 1 | 1 | 1 | 1 | 1 | 0 | 0 | 0 | 1 | 0 | 12 |
| Song S. et al., 2021 | 1 | 1 | 1 | 1 | 0 | 1 | 1 | 1 | 1 | 1 | 1 | 0 | 0 | 1 | 1 | 0 | 12 |
| Stall N.M. et al. 2019 | 1 | 1 | 1 | 1 | 1 | 1 | 1 | 1 | 1 | 1 | 1 | 1 | 1 | 1 | 1 | 0 | 15 |
| Sundvall P.-D., 2015 | 1 | 1 | 1 | 1 | 1 | 1 | 1 | 1 | 1 | 1 | 1 | 1 | 0 | 0 | 1 | 0 | 13 |
| Tandan M., 2018 | 0 | 1 | 1 | 1 | 1 | 0 | 1 | 1 | 1 | 1 | 1 | 0 | 1 | 0 | 1 | 0 | 11 |
| Tandan M., 2019 | 1 | 1 | 1 | 1 | 1 | 0 | 1 | 1 | 1 | 1 | 1 | 0 | 1 | 0 | 1 | 1 | 13 |
| Thompson N.D., 2016 | 1 | 1 | 1 | 1 | 1 | 0 | 1 | 1 | 0 | 1 | 1 | 0 | 0 | 1 | 1 | 0 | 11 |
| Travis L, 2020 | 1 | 1 | 1 | 1 | 1 | 1 | 1 | 1 | 1 | 1 | 1 | 1 | 0 | 1 | 1 | 0 | 14 |
| van Buul L., 2015 | 1 | 1 | 1 | 1 | 0 | 1 | 1 | 1 | 1 | 1 | 1 | 0 | 0 | 0 | 1 | 1 | 12 |
| van der Steen J.T., 2002 | 1 | 1 | 1 | 1 | 1 | 1 | 1 | 1 | 1 | 1 | 1 | 0 | 0 | 1 | 1 | 0 | 13 |
| Vergidis P., 2011 | 1 | 1 | 1 | 1 | 1 | 1 | 1 | 1 | 1 | 1 | 0 | 0 | 0 | 1 | 1 | 0 | 12 |
| Wojkowska-Mach J., 2021 | 0 | 1 | 0 | 1 | 1 | 0 | 0 | 1 | 1 | 1 | 1 | 0 | 0 | 1 | 1 | 0 | 9 |

* Quality of reporting was assessed using a purpose-built standardised tool adapted from the Strengthening the Reporting of Observational Studies in Epidemiology (STROBE) statement checklist.

** 1 if all three biases are addressed; 0 otherwise.

**Table S5** Reference list of the included articles (n=57)

| **Authors** | **Year** | **Journal** | **Title** | **doi** |
| --- | --- | --- | --- | --- |
| Appaneal, H.J et al. | 2021 | J Hosp Infect | Predictors of potentially suboptimal treatment of urinary tract infections in long-term care facilities | 10.1016/j.jhin.2021.01.019 |
| Basso I. and Dimonte V. | 2013 | Assist Inferm Ric | [Nurses' decisions in the care of advanced dementia patients: a survey] | 10.1702/1304.14417 |
| Bennett N et al. | 2018 | Am J Infect Control | Prevalence of infections and antimicrobial prescribing in Australian aged care facilities: Evaluation of modifiable and nonmodifiable determinants | 10.1016/j.ajic.2018.03.027 |
| Bennett N.J. et al. | 2019 | Epidemiol Infect | Skin and soft tissue infections and current antimicrobial prescribing practices in Australian aged care residents | 10.1017/S0950268819000128 |
| Benoit S.R. et al. | 2008 | J Am Geriatr Soc | Factors associated with antimicrobial use in nursing homes: A multilevel model | 10.1111/j.1532-5415.2008.01967.x |
| Boivin Y et al. | 2013 | Med Mal Infect | Antibiotic prescription in nursing homes for dependent elderly people: a cross-sectional study in Franche-Comté | 10.1016/j.medmal.2013.03.004 |
| Brown K.A. et al. | 2020 | Clin Infect Dis | The urine-culturing cascade: Variation in nursing home urine culturing and association with antibiotic use and clostridiodes difficile infection | 10.1093/cid/ciz482 |
| Buehrle D.J. et al. | 2020 | Am J Infect Control | Suprapubic catheter placement improves antimicrobial stewardship among Veterans Affairs nursing care facility residents | 10.1016/j.ajic.2020.01.005 |
| Campitelli M.A. et al. | 2021 | JAMA Netw Open | Comparison of Medication Prescribing before and after the COVID-19 Pandemic among Nursing Home Residents in Ontario, Canada | 10.1001/jamanetworkopen.2021.18441 |
| Cohen C.C. et al. | 2022 | J Am Med Dir Assoc | Examining Nursing Home Information Technology Maturity and Antibiotic Use Among Long-Term Care Residents | 10.1016/j.jamda.2022.01.052 |
| Daneman N. et al. | 2011 | J Antimicrob Chemother | Antibiotic use in long-term care facilities | 10.1093/jac/dkr395 |
| Daneman, N. et al. | 2013 | JAMA Intern Med | Prolonged antibiotic treatment in long-term care role of the prescriber | 10.1001/jamainternmed.2013.3029 |
| Daneman N et al. | 2017 | CMAJ | Influences on the start, selection and duration of treatment with antibiotics in long-term care facilities | 10.1503/cmaj.161437 |
| Durand M. et al. | 2021 | Infect Dis Now | Determinants of doctors' antibiotic prescriptions for patients over 75 years old in the terminal stage of palliative care | 10.1016/j.medmal.2020.10.013 |
| Gouin K.A. et al. | 2022 | Clin Infect Dis | Trends in Prescribing of Antibiotics and Drugs Investigated for Coronavirus Disease 2019 (COVID-19) Treatment in US Nursing Home Residents During the COVID-19 Pandemic | 10.1093/cid/ciab225 |
| Harbin N.J. et al. | 2020 | JAC Antimicrob Resist | Oral and parenteral antibiotic use in Norwegian nursing homes: Are primary care institutions becoming our new local hospitals? | 10.1093/jacamr/dlaa093 |
| Hendricksen M et al. | 2021 | J Am Med Dir Assoc | Factors Associated with Antimicrobial Use in Nursing Home Residents with Advanced Dementia | 10.1016/j.jamda.2020.07.008 |
| Hendriks Simone A. et al. | 2017 | Int J Geriatr Psychiatry | End-of-life treatment decisions in nursing home residents dying with dementia in the Netherlands | 10.1002/gps.4650 |
| Héquet D. et al. | 2021 | J Hosp Infect | Healthcare-associated infections and antibiotic use in long-term care residents from two geographical regions in Switzerland | 10.1016/j.jhin.2021.08.018 |
| Hutt E. et al. | 2008 | J Gerontol A Biol Sci Med Sci | Associations among nurse and certified nursing assistant hours per resident per day and adherence to guidelines for treating nursing home-acquired pneumonia | 10.1093/gerona/63.10.1105 |
| Kistler C.E. et al. | 2017 | J Am Geriatr Soc | The Antibiotic Prescribing Pathway for Presumed Urinary Tract Infections in Nursing Home Residents | 10.1111/jgs.14857 |
| Kistler C.E. et al. | 2020 | J Am Med Dir Assoc | Nursing Home Clinicians' Decision to Prescribe Antibiotics for a Suspected Urinary Tract Infection: Findings From a Discrete Choice Experiment | 10.1016/j.jamda.2019.12.004 |
| Kolodziej L.M. et al. | 2022 | Antibiotics | Resident-Related Factors Influencing Antibiotic Treatment Decisions for Urinary Tract Infections in Dutch Nursing Homes | 10.3390/antibiotics11020140 |
| Loeb M. et al. | 2004 | Infect Control Hosp Epidemiol | Facility-Level Correlates of Antimicrobial Use in Nursing Homes | 10.1086/502373 |
| Mayne S. et al. | 2018 | J Am Geriatr Soc | Confusion Strongly Associated with Antibiotic Prescribing Due to Suspected Urinary Tract Infections in Nursing Homes | 10.1111/jgs.15179 |
| McClean P. et al. | 2011 | Drugs Aging | Antimicrobial prescribing in Nursing Homes in Northern Ireland: Results of two point-prevalence surveys | 10.2165/11595050-000000000-00000 |
| Mehr D.R. et al. | 2003 | Gerontologist | Lower respiratory infections in nursing home residents with dementia: A tale of two countries | 10.1093/geront/43.suppl_2.85 |
| Montgomery P. et al. | 1995 | Journal of Geriatric Drug Therapy | Antimicrobial use in nursing homes in Manitoba | 10.1300/J089V09N03_05 |
| Mylotte J.M. | 1999 | Am J Infect Control | Antimicrobial prescribing in long-term care facilities: Prospective evaluation of potential antimicrobial use and cost indicators | 10.1016/s0196-6553(99)70069-6 |
| Mylotte J.M. and Neff M. | 2003 | Am J Infect Control | Trends in antibiotic use and cost and influence of case-mix and infection rate on antibiotic-prescribing in a long-term care facility | 10.1067/mic.2003.47 |
| Mylotte J.M. and Keagle J. | 2005 | J Am Geriatr Soc | Benchmarks for antibiotic use and cost in long-term care | 10.1111/j.1532-5415.2005.53351.x. |
| Olsho L.E.W. et al. | 2013 | J Am Med Dir Assoc | Does Adherence to the Loeb Minimum Criteria Reduce Antibiotic Prescribing Rates in Nursing Homes? | 10.1016/j.jamda.2013.01.002 |
| Onder G. et al. | 2013 | J Am Med Dir Assoc | Polypharmacy and mortality among nursing home residents with advanced cognitive impairment: Results from the shelter study | 10.1016/j.jamda.2013.03.014 |
| Pengo V. et al. | 2017 | Geriatr Gerontol Int | Advanced dementia: opinions of physicians and nurses about antibiotic therapy, artificial hydration and nutrition in patients with different life expectancies | 10.1111/ggi.12746 |
|  |  |  |  |  |
| Phillips C.D. et al. | 2012 | BMC Geriatr | Asymptomatic bacteriuria, antibiotic use, and suspected urinary tract infections in four nursing homes. | 10.1186/1471-2318-12-73. |
| Pulia M et al. | 2018 | Antimicrob Resist Infect Control | Comparing appropriateness of antibiotics for nursing home residents by setting of prescription initiation: A cross-sectional analysis | 10.1186/s13756-018-0364-7 |
| Quinn K.L. et al. | 2019 | J Gen Intern Med | Association between Physician Intensity of Antibiotic Prescribing and the Prescription of Benzodiazepines, Opioids and Proton-Pump Inhibitors to Nursing Home Residents: a Population-Based Observational Study | 10.1007/s11606-019-05333-8 |
| Raban, M. Z. et al. | 2020 | BMC Health Serv Res | Trends, determinants and differences in antibiotic use in 68 residential aged care homes in Australia, 2014-2017: a longitudinal analysis of electronic health record data | 10.1186/s12913-020-05723-3 |
| Roughead E.E. et al. | 2009 | Med J Aust | Proton-pump inhibitors and the risk of antibiotic use and hospitalisation for pneumonia | 10.5694/j.1326-5377.2009.tb02307.x. |
| Roukens M. et al. | 2017 | J Antimicrob Chemother | Surveillance of antimicrobial use in Dutch long-term care facilities | 10.1093/jac/dkw581 |
| Rummukainen M.-L. et al. | 2013 | Infection | Antimicrobial prescribing in nursing homes in Finland: Results of three point prevalence surveys | 10.1007/s15010-012-0331-9 |
| Saxena Farah E. et al. | 2019 | J Am Geriatr Soc | The Association of Resident Communication Abilities and Antibiotic Use in Long-Term Care | 10.1111/jgs.15771 |
| Sloane P.D. et al. | 2017 | Infect Control Hosp Epidemiol | Urine culture testing in community nursing homes: Gateway to antibiotic overprescribing | 10.1017/ice.2016.326 |
| Sluggett J.K. et al. | 2021 | J Antimicrob Chemother | Contribution of facility level factors to variation in antibiotic use in long-Term care facilities: A national cohort study | 10.1093/jac/dkab007 |
| Smith C.M. et al. | 2020 | BMC Health Serv Res | Antibiotic prescribing in UK care homes 2016-2017: retrospective cohort study of linked data | 10.1186/s12913-020-05422-z |
| Sommer-Larsen S.D. et al. | 2021 | Antibiotics | Quality of the diagnostic process, treatment decision, and predictors for antibiotic use in general practice for nursing home residents with suspected urinary tract infection | 10.3390/antibiotics10030316 |
| Song S. et al. | 2021 | J Am Geriatr Soc | Antibiotic Use Among Residents Receiving Skilled Nursing Care in 29 U.S. Nursing Homes | 10.1111/jgs.16856 |
| Stall N.M. et al. | 2019 | JAMA Netw Open | Sex-Specific Differences in End-of-Life Burdensome Interventions and Antibiotic Therapy in Nursing Home Residents with Advanced Dementia | 10.1001/jamanetworkopen.2019.9557 |
| Sundvall P.-D. et al. | 2015 | BMC geriatrics | Antibiotic use in the care home setting: a retrospective cohort study analysing routine data | 10.1186/s12877-015-0073-5 |
| Tandan, M. et al. | 2018 | Euro Surveill. | Antimicrobial prescribing and infections in long-term care facilities (LTCF): A multilevel analysis of the HALT 2016 study, Ireland, 2017 | 10.2807/1560-7917.ES.2018.23.46.1800278 |
| Tandan, M. et al. | 2019 | J Am Med Dir Assoc | Improving Antimicrobial Prescribing: A Multinomial Model Identifying Factors Associated With First- and Second-Line Prescribing | 10.1016/j.jamda.2018.10.028 |
| Thompson, N.D. et al. | 2016 | J Am Med Dir Assoc | Prevalence of Antimicrobial Use and Opportunities to Improve Prescribing Practices in U.S. Nursing Homes | 10.1016/j.jamda.2016.08.013 |
| Travis L. et al. | 2020 | Infect Control Hosp Epidemiol | Burden of antibiotic allergy labels in Australian aged care residents: Findings from a national point-prevalence survey | 10.1017/ice.2020.53 |
| van Buul L. et al. | 2015 | J Am Med Dir Assoc | Antibiotic prescribing in dutch nursing homes: How appropriate is it? | 10.1016/j.jamda.2014.10.003 |
| van der Steen et al. | 2002 | Arch Intern Med | Withholding antibiotic treatment in pneumonia patients with dementia | 10.1001/archinte.162.15.1753 |
| Vergidis P et al. | 2011 | J Am Geriatr Soc | Patterns of antimicrobial use for respiratory tract infections in older residents of long-term care facilities | 10.1111/j.1532-5415.2011.03406.x. |
| Wojkowska-Mach J. et al. | 2021 | Antimicrob Resist Infect Control | Antibiotic consumption in long-term care facilities in Poland and other European countries in 2017 | 10.1186/s13756-021-01019-1 |

**Table S6** Summary of the characteristics of the included studies (n=57)

| **Characteristics** | **Number of studies** |
| --- | --- |
| **Study country*** |  |
| USA | 21 |
| Canada | 10 |
| Australia | 7 |
| Netherlands | 6 |
| UK | 3 |
| France | 2 |
| Ireland | 2 |
| Several European countries | 2 |
| Denmark | 1 |
| Finland | 1 |
| Italy | 2 |
| Norway | 1 |
| Switzerland | 1 |
| **Study year (last year of data collection)** |  |
| 1987 to 2000 | 5 |
| 2001 to 2005 | 5 |
| 2006 to 2010 | 6 |
| 2011 to 2015 | 16 |
| 2016 to 2020 | 25 |
| **Study design** |  |
| Longitudinal study | 34 |
| Cross-sectional study | 20 |
| Ecological study | 1 |
| Discrete Choice Experiment or case study | 2 |
| **Follow-up duration (for longitudinal studies)** |  |
| Less than 1 month | 2 |
| 1 to 6 months | 11 |
| 7 to 12 months | 9 |
| More than one year | 9 |
| N/R | 4 |
| **Study population** |  |
| LTCF residents without any specificity | 36 |
| LTCF residents with specific infections | 11 |
| LTCF residents with dementia | 7 |
| Physicians | 3 |
| **Type of outcome** |  |
| Overall volume of antibiotic use only | 45 |
| Inappropriateness only | 10 |
| Both types of outcomes | 2 |
| **Type of factors investigated** |  |
| Resident and facility and prescriber-level | 3 |
| Resident and facility-level | 12 |
| Resident-level only | 14 |
| Facility-level only | 17 |
| Prescriber-level only | 5 |
| Other cases | 6 |
| **Factors associated with antibiotic use** |  |
| Principal objective | 20 |
| Secondary objective | 37 |
| **Statistical analysis** |  |
| Unadjusted analysis only | 22 |
| Adjusted analysis | 35 |
| **Reporting quality score (/16)** |  |
| 7 to 10 | 11 |
| 11 (Q1) | 9 |
| 12 (median) | 12 |
| 13 (Q3) | 12 |
| 14 or 15 | 13 |

* One study included data from the USA and from Canada and one study included data from the USA and the Netherlands.

LTCF: long-term care facility; N/R: not reported; USA: United States of America
